# Study of Displacement Efficiency and Flow Behavior of Foamed Gel in Non-Homogeneous Porous Media

**DOI:** 10.1371/journal.pone.0128414

**Published:** 2015-06-01

**Authors:** Yanling Wang, Jiafeng Jin, Baojun Bai, Mingzhen Wei

**Affiliations:** 1 Petroleum Engineering College, China University of Petroleum (East China), Qingdao Shandong, P.R. China; 2 Department of Geological Science and Engineering, Missouri University of Science and Technology, 1400 N. Bishop Avenue, Rolla, Missouri, United States of America; Tianjin University, CHINA

## Abstract

Field trials have demonstrated that foamed gel is a very cost-effective technology for profile modification and water shut-off. However, the mechanisms of profile modification and flow behavior of foamed gel in non-homogeneous porous media are not yet well understood. In order to investigate these mechanisms and the interactions between foamed gel and oil in porous media, coreflooding and pore-scale visualization waterflooding experiments were performed in the laboratory. The results of the coreflooding experiment in non-homogeneous porous media showed that the displacement efficiency improved by approximately 30% after injecting a 0.3 pore volume of foamed gel, and was proportional to the pore volumes of the injected foamed gel. Additionally, the mid-high permeability zone can be selectively plugged by foamed gel, and then oil located in the low permeability zone will be displaced. The visualization images demonstrated that the *amoeba effect* and Jamin effect are the main mechanisms for enhancing oil recovery by foamed gel. Compared with conventional gel, a unique benefit of foamed gel is that it can pass through micropores by transforming into arbitrary shapes without rupturing, this phenomenon has been named the *amoeba effect*. Additionally, the stability of foam in the presence of crude oil also was investigated. Image and statistical analysis showed that these foams boast excellent oil resistance and elasticity, which allows them to work deep within formations.

## Introduction

Foamed gel has been applied broadly as a mobility-control and profile-modification agent for enhanced-oil-recovery (EOR) Process in the petroleum industry. Compared with conventional foam, foamed gel possesses excellent stability, oil resistance, and elasticity, which makes it a good candidate for profile modification treatments [1–4]. Previous studies showed that oil recovery could be improved by 4.68%~47.88% after foamed gel treatment [5]. However, few studies have investigated the relationship between the displacement efficiency and pore volume of fluid injected, and the mechanisms of enhanced-oil-recovery and flow behavior of foamed gel in porous media are not yet well understood.

In 1995, Matthew et al. [6] noted that foamed gel may be used to plug porous media for the purpose of physically controlling the movement of subsurface fluids, and the adsorption and trapping effect of foamed gel can effectively shut off the high-permeability zone. Miller and Fogler [7]demonstrated that in-situ generated foamed gel barriers were well suited for long-term waterflooding diversion because they provided an intermediate degree of plugging, yet required substantially less polymer than bulk gels. Wassmuth et al. [8] stated that foamed gel exhibited better gas blocking capability, a greater effective lifetime, and larger residual resistance factors than conventional foams at a temperature of 85°C. Foamed gel is suitable for the following three potential applications:1) mobility control, to improve the displacement efficiency of waterflooding; 2) partial or total pore plugging, which regulates the flow condition of fluids and plugs higher permeability formations; and 3) increasing sweep efficiency by expanding the swept volume of the reservoir [9,10].

Those above studies illustrate that foamed gel can serve as an effective method by which to enhance oil recovery. However, less research has been done on the displacement efficiency of foamed gel in non-homogeneous reservoirs, and many mechanisms governing the flow behavior of foamed gel and the interactions between foamed gel and crude oil are not yet well understood. The investigation presented in this paper has two experimental goals 1) to demonstrate that foamed gel is suitable for enhancing oil recovery in non-homogeneous reservoirs, and 2) to research the flow behavior of foamed gel when oil is present in porous media by using a microvisual model. The characteristics of foamed gel also are discussed.

## Materials

### Materials

**Fig 1:**
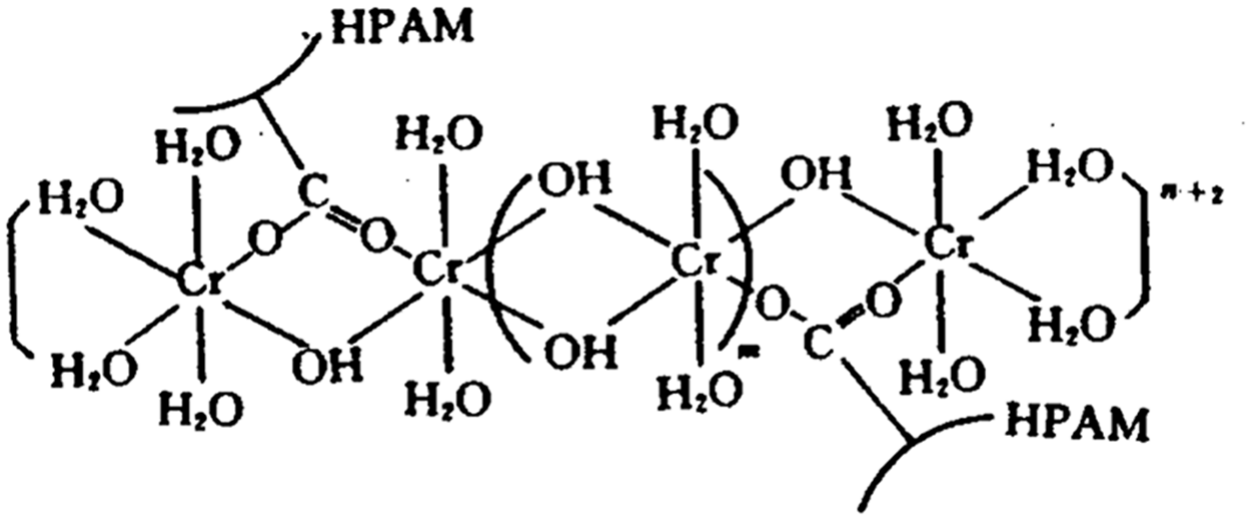
Structure of the formula after gelation.

The materials used in this study included a Sony 79 video digital camera; 5×5cm micromodel; HH temperature-controlled bath; XJ-7 plunger pump; 101A-1E blast electric oven; and electronic balance. Fig 1 shows a schematic of the foamed gel flow experimental set-up. The micromodel was characterized by macroscopic heterogeneities, with a random network of large pores that spanned the micromodel while being highly interconnected with a random network of small pores.

All displacement tests were carried out using synthetic brine having a total dissolved solid (TDS) content of 3.42wt%. The composition of the synthetic brine is presented in Table 1.

**Table 1:** Synthetic brine composition.

Components	w/w %
NaCl	2.73
MgCl_2_:6H_2_O	0.15
CaCl_2_	0.38
Na_2_SO_4_	0.16

The polymer used in this study were partially hydrolyzed polyacrylamide (HPAM), offered by Liaohe Oilfield; Crosslinking agent, offered by Liaohe Oilfield; NaNO_2_, an analytical reagent offered by Sinopharm; NH_4_Cl, an analytical reagent offered by Sinopharm; Sodium dodecyl sulfonate (SDS), a foaming agent offered by Liaohe Oilfield; Distilled water; N-decane, an analytical reagent offered by the sinopharm; And methylene blue methythionine chloride, a chemical reagent offered through the work of Hedong Tianjin.

Crude oil and cores used in this experimental study, offered by Liaohe oilfield. The density and viscosity of the crude oil were 0.928 gr/cm^3^ and 56mPa·s at25°C, respectively; The cores used in the experiments can be seen in Table 2.

**Table 2:** Core data.

NO	L(cm)	Cross-sectional area(cm^2^)	K (md)
120227A-1	61.10	19.71	2882
120227A-2	61.10	19.74	3106
120227A-3	61.00	19.72	2986
120227A-4	61.10	19.49	3224
120227A-5	61.00	19.75	2725
120228A-1	61.00	19.90	1509
120228A-2	60.60	19.48	1477
120228A-3	60.60	19.65	1500
120228A-4	60.65	19.64	1520
120228A-5	60.65	19.65	1573
120229A-1	60.70	19.59	215
120229A-2	60.70	19.57	248
120229A-3	60.60	19.53	236
120229A-4	60.60	19.47	210
120229A-5	60.60	19.65	264

## Experimental Methods

### Foamed gel preparation

The foamed gel used in this study was prepared according to the following procedures. Polymer solution was mixed with cross-linker, and then NaNO_2_, NH_4_Cl, and SDS were added into the solution. The mixture was fully stirred before being injected into the core. Polymer and cross-linker can improve the stability of foam by forming a gel film on the foam surface. The reaction of NaNO_2_ and NH_4_Cl can generate N_2_, SDS as a foaming agent.

The formulation compositions were as follows: Polymer 0.2wt%; cross-linker 0.2wt%, NaNO_2_ 0.2wt%, NH_4_Cl 0.3wt%, SDS 1.0wt%.

N_2_ was produced according to the following chemical equation, and the structure of the crosslinked gel appears in Fig 1.

### (1) $NaNO_{2}+NH_{4}Cl\overset{H+}{\to }N_{2}↑+NaCl+2H_{2}O$ Determination the breakthrough-vacuum of foamed gel

**Fig 2:**
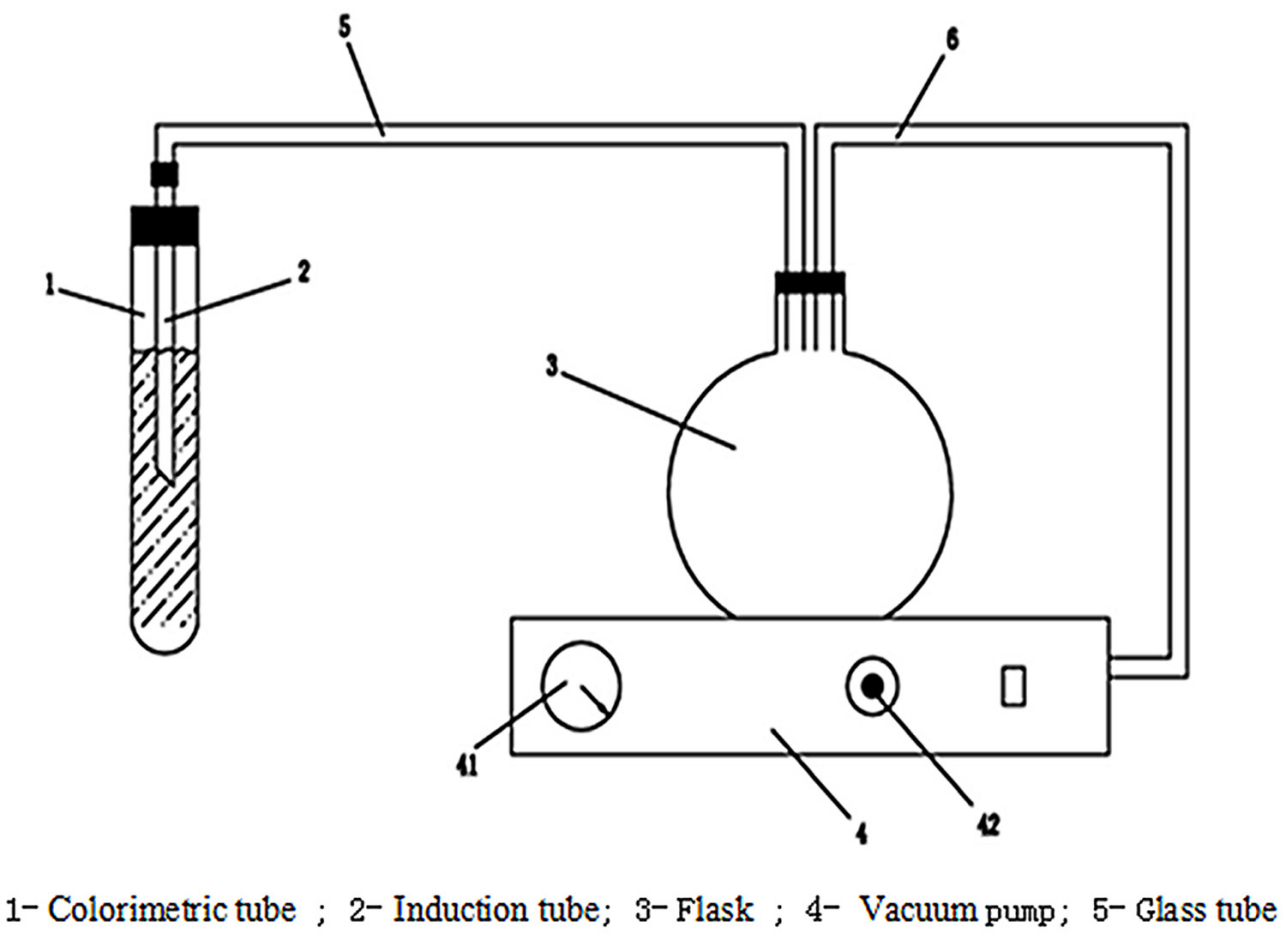
Schematic of breakthrough-vacuum apparatus.

The gel strength of foamed gel was measured using the breakthrough-vacuum method. Approximately 25 mL of foamed gel was transferred to a colorimetric tube; after deaeration, it was kept in a water bath at a constant temperature for 48 hours to obtain foamed gel. The glass tube, which was connected to a vacuum pump, was quickly put into the gel, and the largest value on the vacuum meter (the breakthrough vacuum) was written down. Every sample was measured for three times, and the average value was used as the gel strength for the sample [11]. The schematic of the breakthrough-vacuum apparatus appears in Fig 2.

### (2) Coreflooding

Coreflooding tests were conducted to evaluate the displacement efficiency and superior shut-off capacity of foamed gel in non-homogeneous porous media. The three cores simulating interlayer heterogeneity were used to improve oil recovery, foamed gel as a plugging agent. Both cores were 60cm long, and the inside diameter was 2.50cm. Firstly, the cores were saturated with synthetic brine for 8 hours, and then a drainage process followed using crude oil until the residual saturation of brine was reached. In the third step, the core was flooded by brine until the water-cut of the production fluid increased to 98%. The foaming solutions eventually would transform into foamed gel in the micromodel, which could generate foam. After the foamed gel was injected, the cores were shut in for 48 hours for gelation. After gelation, water was injected at a constant rate, and the total amount of liquid during the displacement was recorded by a computer. The injection rate was 0.5ml/min.

### (3) Visualization of flooding

**Fig 3:**
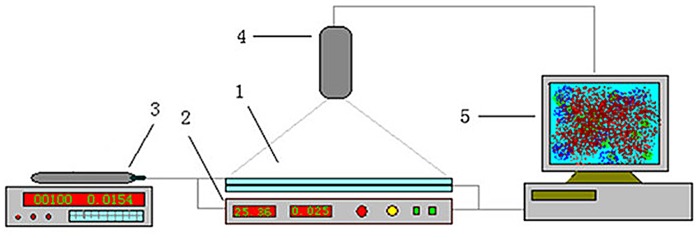
Schematic of micromodel apparatus. 1-Micromodel; 2-Bottom light physical model chamber (iosthermal controllable); 3-Micro pump; 4-Video camera; 5-Computer.

**Fig 4:**
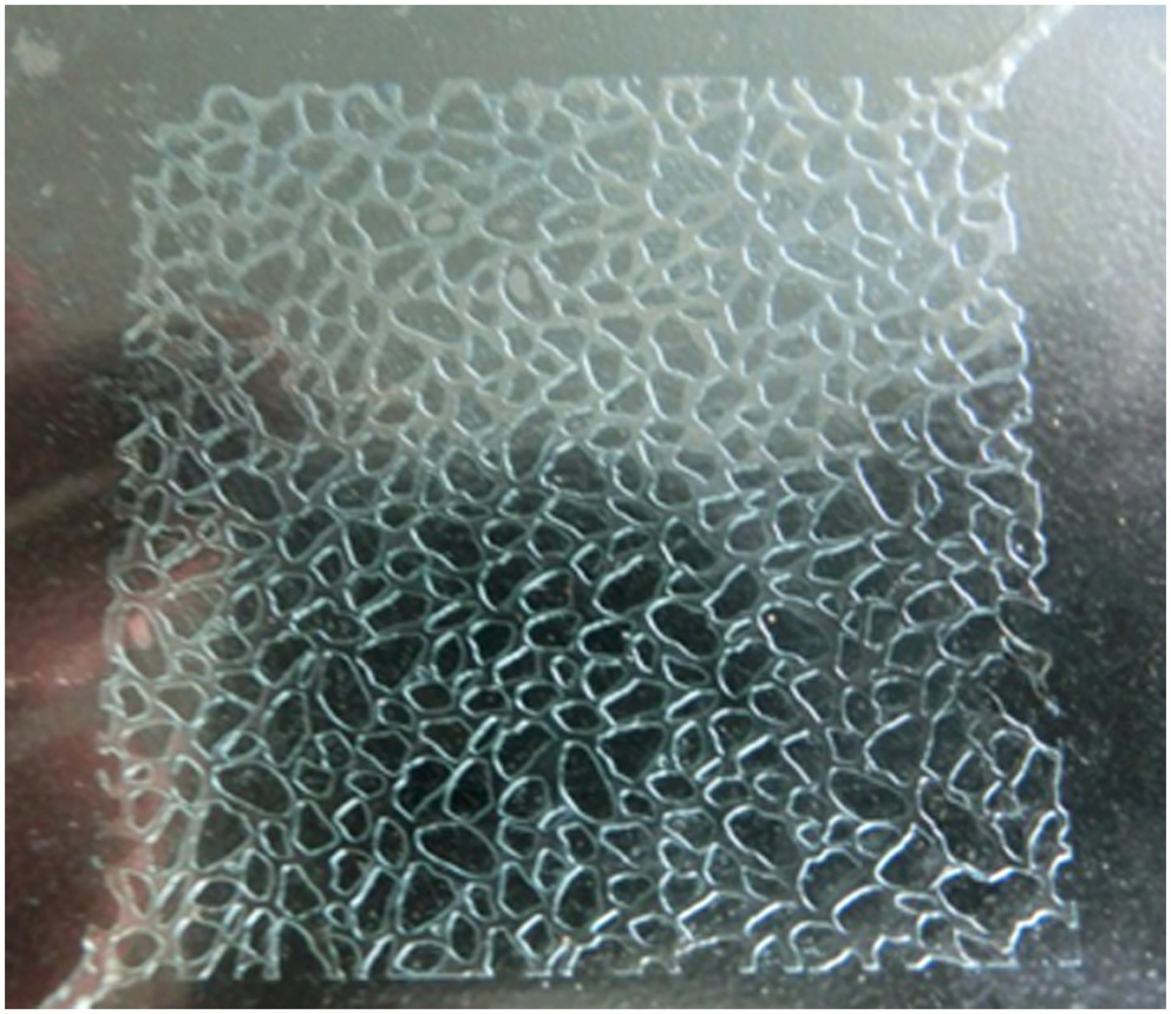
Etched glass micromodel.

The experiment was performed using the apparatus shown in Figs 3 and 4 at room temperature and under strongly water-wet conditions. The procedures were as follows:

1. The micromodel was saturated with synthetic brine for 8 hours.
2. A drainage process followed using crude oil until the residual saturation of brine was reached.
3. The visualization waterflooding was conducted until the residual oil saturation was reached.
4. Foaming solutions were mixed together and fully stirred before being injected into the micromodel. These foaming solutions eventually would transform into foamed gel in the micromodel. In order to guarantee the displacement effect of the foamed gel displacement, the micromodel was shut in for 48 hours for gelation.
5. After gelation, water was injected at a fixed flow rate to observe the flow behavior of foamed gel and the interactions between the foamed gel and the crude oil in porous media.

The injection rate was controlled by a micro-pump, a digital video camera was used to record the flow behavior of the foamed gel in the micromodel. Each injection step was videotaped and photographed for further image analysis.

## Results and Discussion

### Strength and viscosity of foamed gel

**Fig 5:**
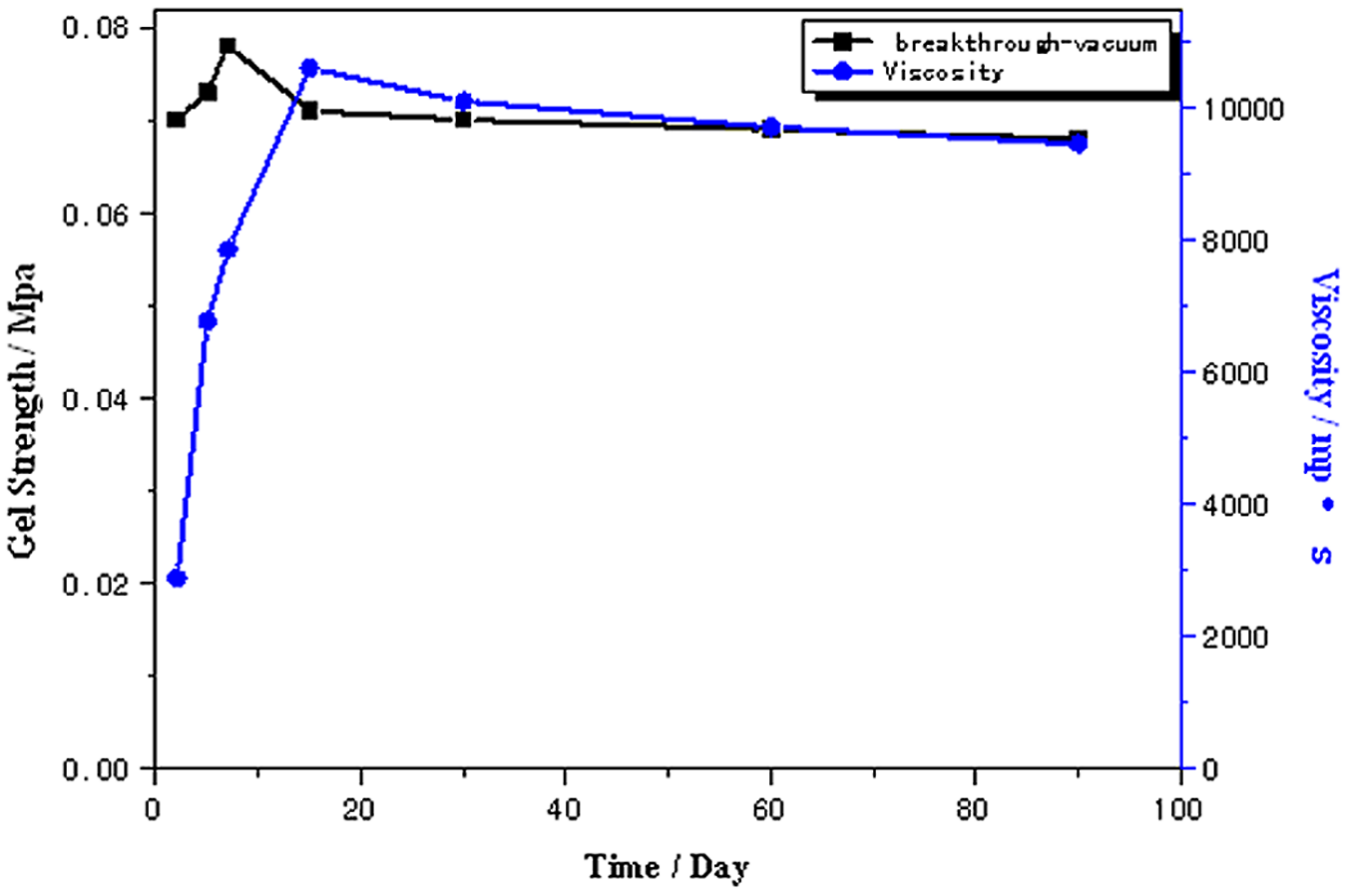
Gel strength and viscosity as a function of gelation time.

Fig 5 illustrates the foamed gel strength as a function of time at room temperature. The viscosity of foamed gel is one of key factors to maintain the stability of foam. The cross-linking process of foamed gel took over 90 days. As can be seen from Fig 5, the strength of foamed gel reached to 0.07MPa after 2 days (48 hours), the viscosity of foamed gel increased from 2000mp·s to approximately 4000 mp·s, which was considered as a strong foamed gel. As gelation time increased to 15 days (288 hours), this foamed gel reached its highest strength, forming a highly deformable, non-flowing foamed gel, thus foamed gel could enter into deep formation to profile before gelation. Therefore, foamed gel shows good injectivity to the formation. In these experiments, the micromodel was shut in for 48 hours for gelation reaction after the foamed gel was placed within the micromodel before resuming the injection of gas and water.

### Coreflooding tests

**Fig 6:**
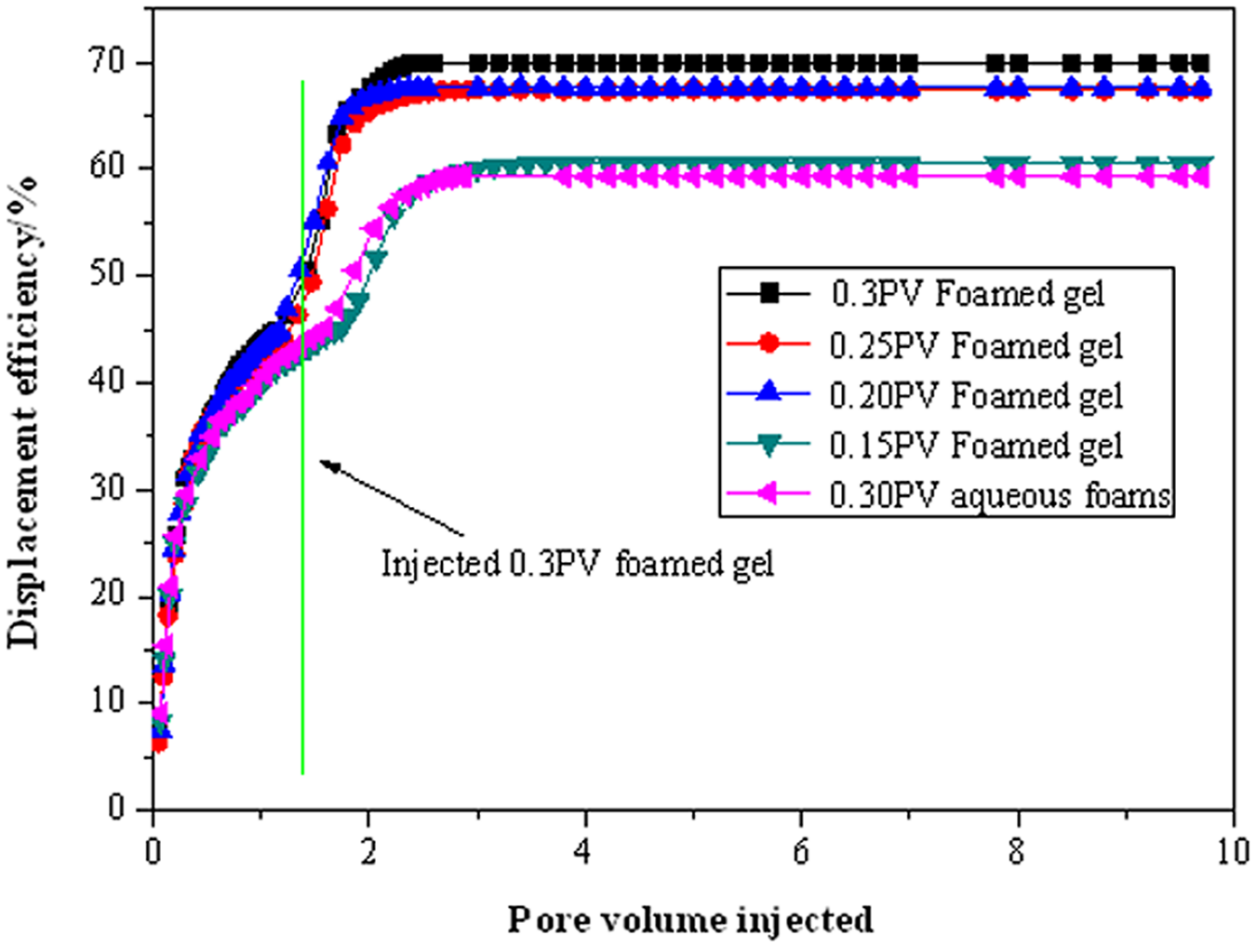
Displacement efficiency as a function of pore volume of fluid injected.

Coreflooding were conducted in three cores simultaneously. Core1, core2, and core3 were high-permeability, mid-permeability, and low-permeability cores, respectively. Fig 6 shows the results of the displacement efficiency after 0.15PV, 0.2PV, 0.25PV, and 0.3PV of foamed gel were injected. For the purpose of comparison, 0.3PV of aqueous gel also was injected into the cores. In the first stage, the displacement efficiency of waterflooding was approximately 40%; During continuous waterflooding over 1.35PV, oil was produced mainly from the mid-to high-permeability cores, and oil recovery from the low-permeability core was nearly zero. In the second stage, the displacement efficiency improved significantly to approximately70% after injecting 0.3PV of foamed gel. The displacement efficiency after injecting an equivalent volume of aqueous gel was only about 58%. When the pore volume of injected fluid reached approximately 4, the displacement efficiency of the different pore volumes injected tended to stabilize. In this stage, oil was produced mainly from the low-permeability cores, the mid- to high-permeability cores had been selectively plugged, produced a small amount of oil. Additionally, SDS of foamed gel can emulsify crude oil through the interaction with it, which significantly decrease the viscosity resistance of crude oil, the flow condition of crude oil could be improved. Therefore, foamed gel can be used to enhance oil recovery by selectively plugging and emulsification in non-homogeneous reservoirs.

**Fig 7:**
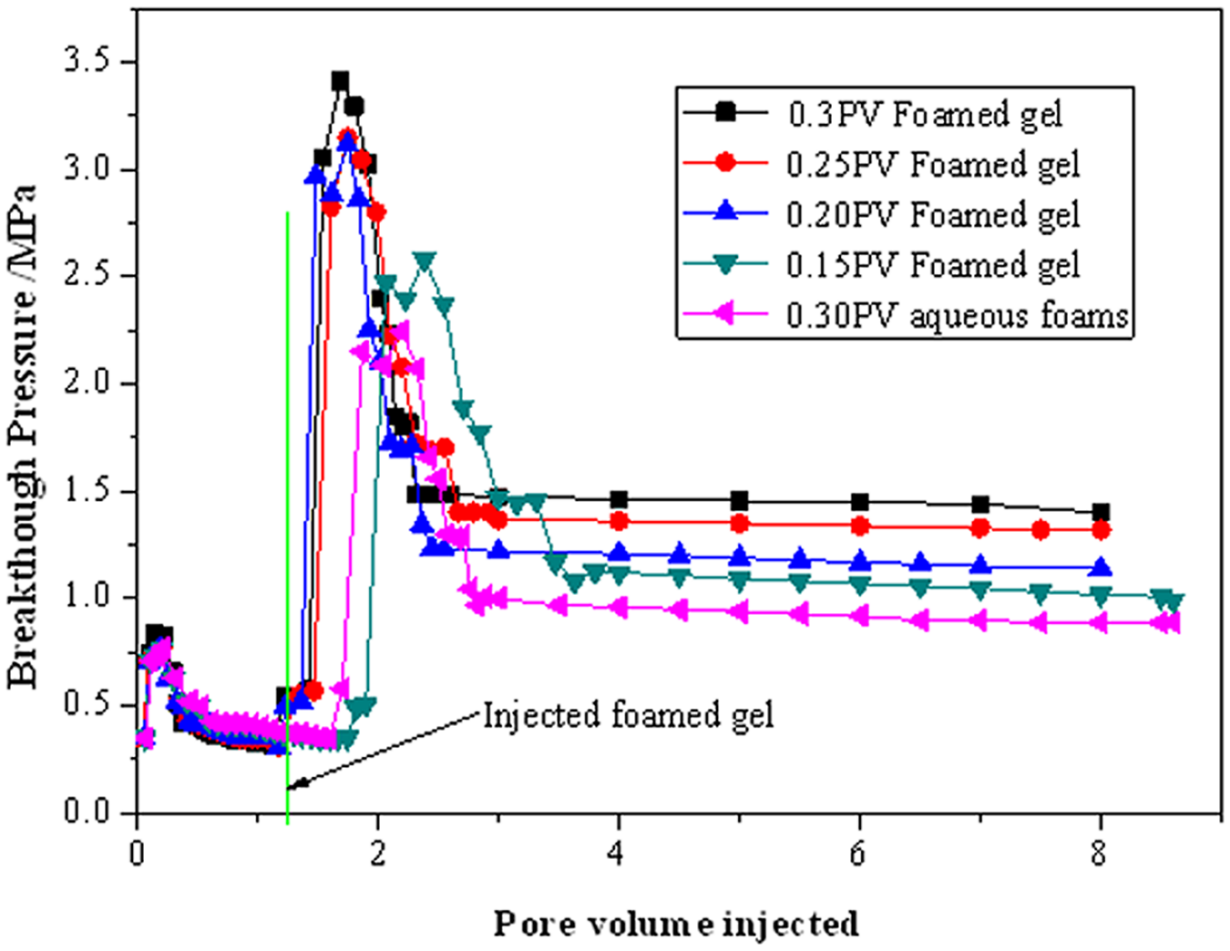
Breakthrough Pressure as a function of pore volume of fluid injected.

**Fig 8:**
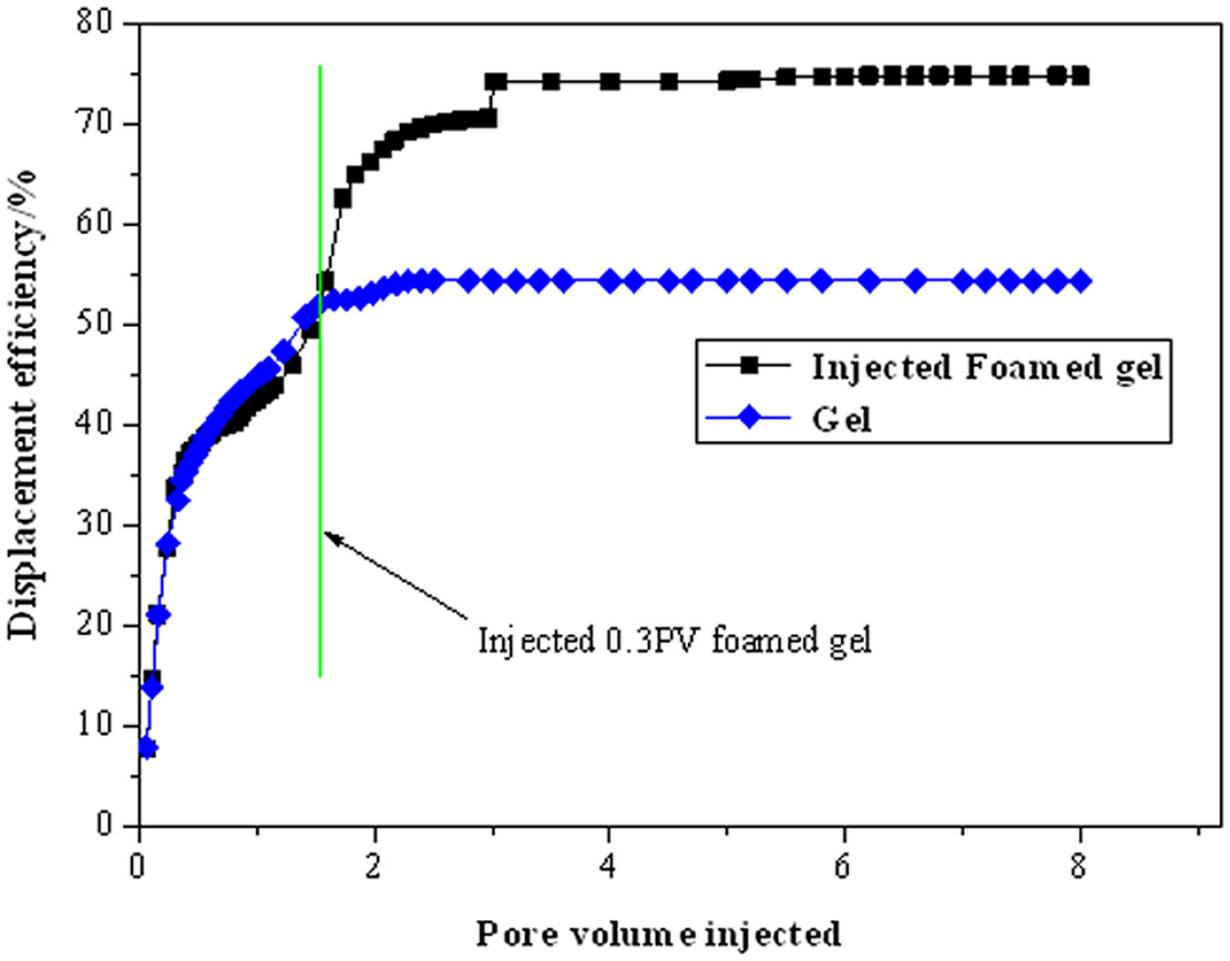
Comparison of foamed gel with conventional gel.

Fig 7 shows the breakthrough pressure as a function of pore volume of fluid injected was recorded during the displacement process. As can be seen from Fig 7, the breakthrough pressure increased from 0.3Mpa to approximately 3.5MPa after injecting foamed gel. Moreover, the breakthrough pressure after injecting 0.3PV of foamed gel was clearly much higher than that injecting less than 0.3PV of foamed gel, and was more than 60% of that after injecting 0.3PV of aqueous foam. As the pore volume of injected fluid reached to 4, the displacement efficiency was close to 70%, as presented in Fig 8. The results indicate that the mid- to high-permeability cores were selectively plugged by foamed gel, which result in a much higher starting pressure switch to the low-permeability core, so fluid was mainly produced from the low-permeability core. Meanwhile, the breakthrough pressure response depended significantly on the volume of foamed gel injected into the cores, the breakthrough pressure was proportional to the injected volume of foamed gel, and the flow of foamed gels generated higher breakthrough pressure than the flow of aqueous foam because of the higher viscosity of foamed gels [12] and the texture of foamed gel may enhance its stability in the porous media.

Fig 8 shows the displacement deficiency after injecting foamed gel and conventional gel, respectively. The displacement deficiencies of the low-mid-high permeability cores before foamed gel injection were 38.36%, 16.18%, and 7.4%, respectively. Oil was produced mainly from the mid- to high-permeability cores before foamed gel treatment, but the production potential of the low-permeability core increased by 51.78% after injecting foamed gel, as listed in Table 3. Therefore, foamed gel is obviously superior to conventional gel for enhancing oil recovery in non-homogeneous reservoirs.

**Table 3:** Oil displacement test by injecting 0.3PV of foamed gel.

Core	Porosity/%	K/μm^2^	S_oi_/%	Displacement efficiency before treatment/%	Displacement efficiency after treatment /%
1	27.18	3.162	97.5	38.36	56.17
2	25.48	0.902	96	16.18	64.71
3	23.78	0.258	78.6	7.4	51.78

## Visualization of Waterflooding in the Micromodel

### Microscopic waterflooding

**Fig 9:**
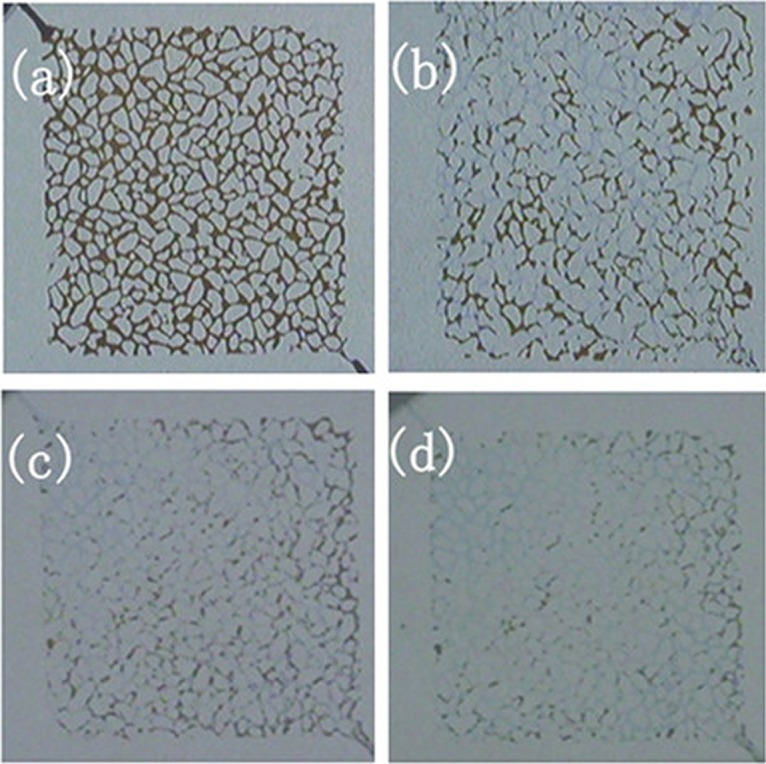
Visualized waterflooding. (a) Saturated by crude oil; (b) Displacing to residual saturation; (c) Before foamed gel treatment; (d) After foamed gel treatment.

Fig 9 exhibits a series of photographs of fluid distributions in the micromodel before and after injecting foamed gel. The darkest sections correspond to crude oil, and the intermediate grey areas represent the injected solution.

Fig 9a shows that the micromodel was saturated with crude oil to the initial oil saturation. Fig 9b shows a photograph of the pore network model at the residual oil saturation before injecting foamed gel, it can clearly be seen that lots of irreducible oil can’t be swept, still occupy the pore throat of micromodel. After foamed gel injection, the micromodel was shut in for 48 hours for gelation. According to Fig 9d, foamed gel plugged water advantage channel of micromodel, more than 90% of the residual oil in unswept region was displaced from the micromodel, the displacement efficiency could be improved greatly after injecting foamed gel.

### Oil resistance of foamed gel

**Fig 10:**
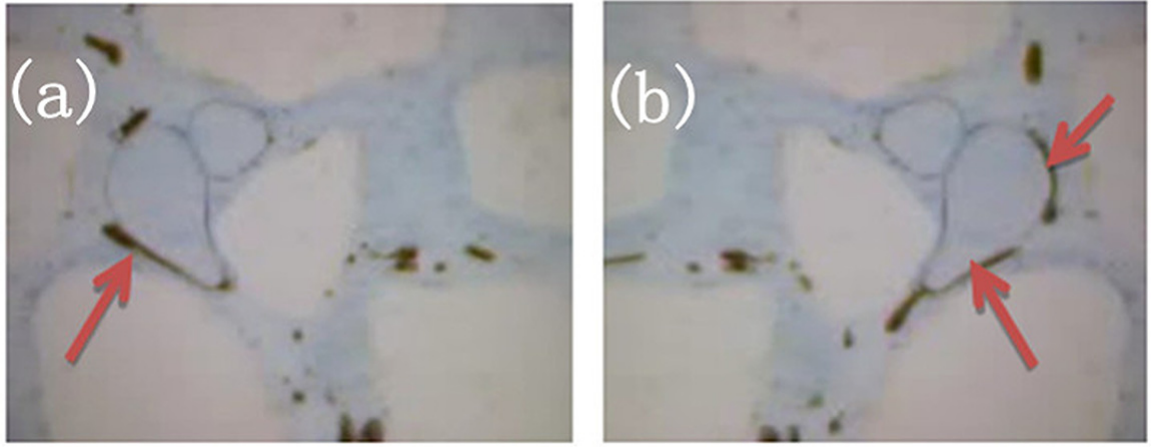
Good oil resistance of foamed gel(magnified 300x). (a) Foaming trapping; (b) Oil film on foam surface.

Oil resistance is one of the key capacities in maintaining the stability of foamed gel in formation. The foams generated by foamed gel did not rupture when they encountered crude oil in the porous media. As shown in Fig 10b, an oil droplet could transform into a thin oil film on the foam surface because under the interaction between water and foamed gel. However, the large foam didn’t rupture because that the gel film on the foam surface enhanced the mechanical stability of the foam system. Additionally, primarily in intermediate-sized pores, the foams can become trapped around these pores, fluid diversion generated by these trapped foams led more fluid flowing to unswept region. Fig 10a shows the small pore throat is occupied by the aqueous liquid, and the large pore throat carry the flowing foam. After gelation reaction, minute foams can serve as the displacing phase to displace crude oil in the smallest pore. However, due to the viscous resistance, large foams will selectively plug large pores to regulate the flow direction of fluid in pore throat. In this case, foamed gel can be applied effectively to solve the problem of high-water-cut oil-producing wells in non-homogeneous reservoirs [13–17].

### Amoeba effect of foamed gel

**Fig 11:**
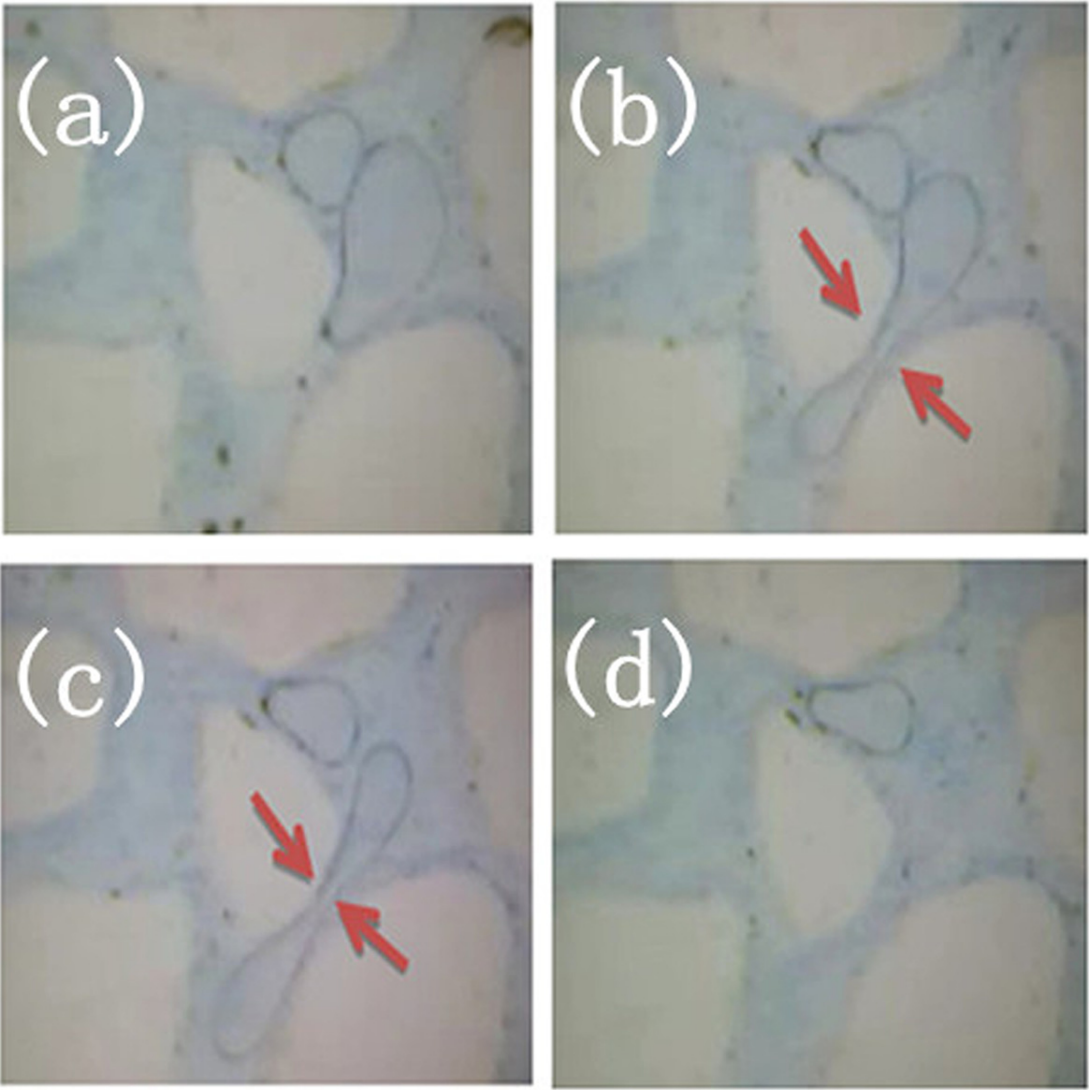
Amoeba effect I. (a) Foamed gel distribution in micromodel; (b) Dumbbell-shaped foam; (c) Dumbbell-shaped foam; (d) Foamed gei distribution after placement.

**Fig 12:**
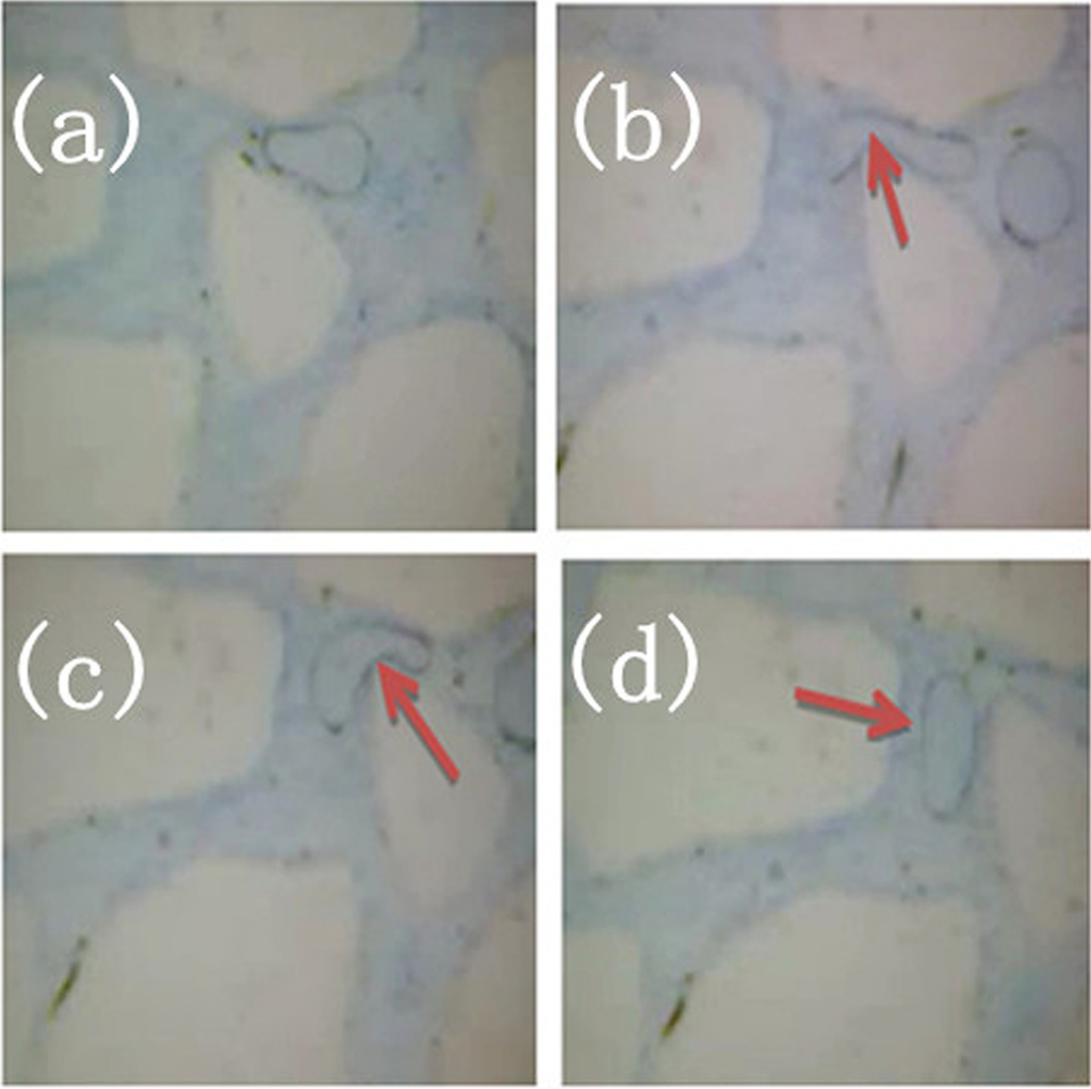
Amoeba effect II. (a) The foam starts to go through the pore throat; (b) The foam is passing through the pore throat; (c) The foam is passing through the pore throat; (d) The foam has totally passed through the pore throat.

Fig 11 represents the deformation behavior of foam located in the small pore throat during the displacement process. The major pore throats were filled with foam, while the pore bodies contained gel solution. The fluid acted as a displacing force to facilitate foam passing through the pore throats. Fig 11a shows a section of the micromodel containing two continuous foamed gel texture configurations, one of the foamed gel was attempting to pass through a narrow pore throat. Fig 11b, which corresponds to the flow behavior of foamed gel, it was quite clear that large foam passed through a narrow pore throat by deforming into slender dumbbell-shaped foam with large ends and slim middle. In the process of passing through the pore throat, the middle part of the foam remained stable without rupture; this phenomenon was named the *amoeba effect*. A reasonable explanation is that the external phase of foam is cross-linked gel film, thus the strength of the foam is significantly enhanced, which guarantees that the foams can pass through most pore throats without rupture; Therefore, foamed gel can enter into deep formation to modify the profile. Compared with foamed gel, the external surfaces of conventional foams do not have a gel film, so the stability of these conventional foams is not so good, they easily rupture when passing through small pore throats. As shown in Figs 12a and 12b, the small foam attempted to pass through the pore throat at nearly 90°. Fig 12c reveals the excellent bending deformation of foamed gel, in order to pass through the pore, the foamed gel acted like an amoeba by deforming into another shape on the pore wall. The flow behaviors of foamed gel are different from conventional gel. Generally, the flow resistance of gel comes from interlayer friction, while the flow resistance of foamed gel comes from inter layer friction and additional extruded force generated by the Jamin effect. Meanwhile, owing to the transformation of the foams when they pass through pore throats, the additional extruded force increases, and the oil absorbed on the pore walls can be displaced clearly. During the process of flooding, foamed gel firstly enters into the large pores whose flow resistance is smaller than that of the small pores. The pore space previously occupied by oil or water now is taken over by foamed gel. The viscosity resistance of foamed gel is larger than that of the fluid, so the follow-up water flooding can only pass through the mid- to low-permeability zones, which have more oil and have not been occupied by foamed gel. And then, the residual oil remaining in unswept region can be displaced by foamed gel.

### Jamin effect of foamed gel

**Fig 13:**
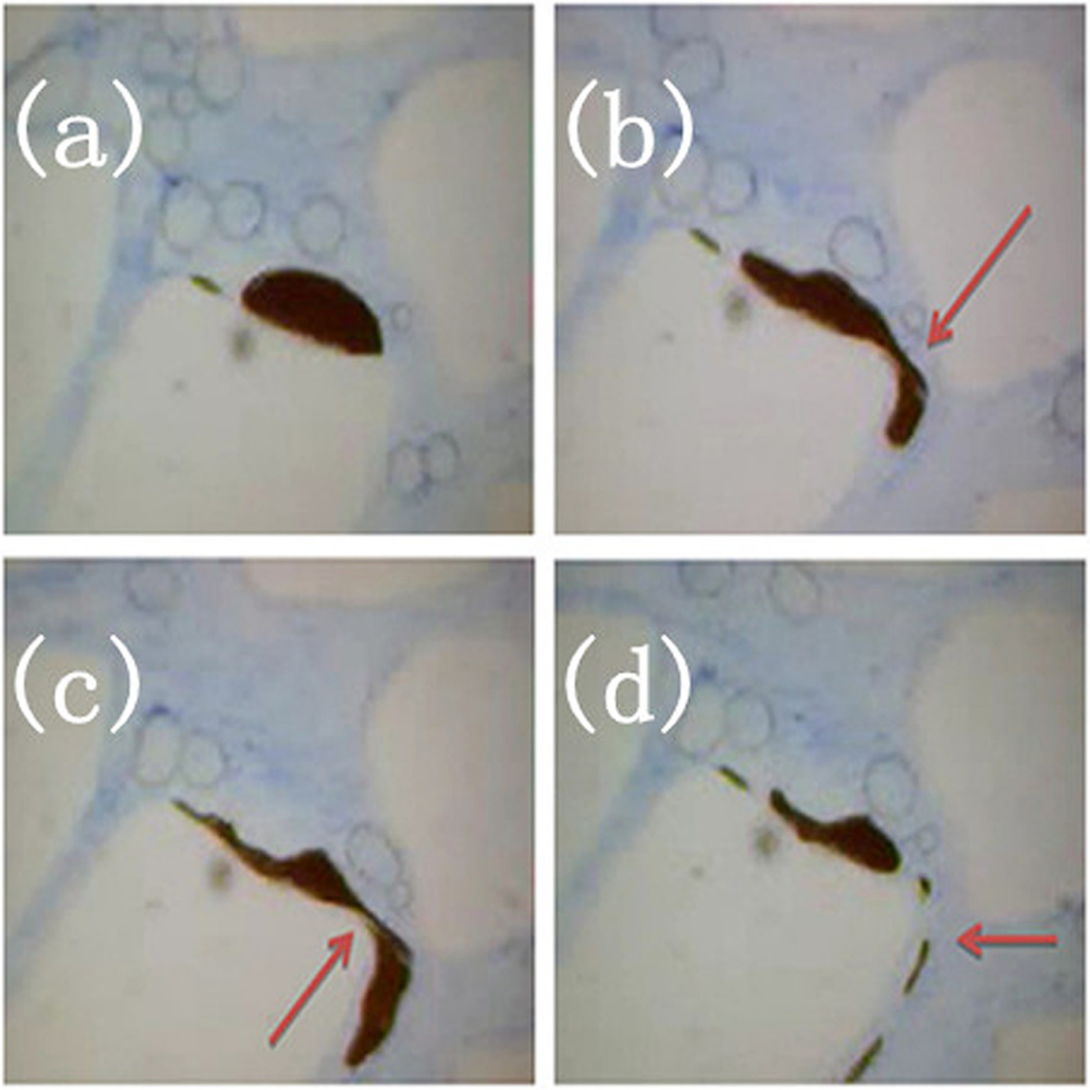
Jamin effect of foamed gel. (a) Foam try to pass through the throat; (b) Jamin effect; (c) Jamin effect; (d) Oil passing through the throat.

The above figures present that the pore throats are occupied by a large amount of foam. If the amount of foams reaches a certain level as the fluid migrates continuously, much of the foam will come together and generate great additional force to crude oil; this additional force can be transformed into displacing force. In Fig 13a, foamed gel begins to extrude crude oil during the process of flooding. Due to the increasing additional displacing force generated by the foam, the oil droplet will break into small pieces to pass through the pore throat, as shown in Fig 13b and 13c demonstrates the critical state of the oil droplet passing through the pore throat. As fluid flows in porous media, large oil droplets gradually break into small pieces; Meanwhile, the flow of foams can increase the displacement efficiency significantly. Additionally, the surfactant of the foamed gel can emulsify crude oil, which also can improve displacement efficiency.

## Conclusion

1. The strength and viscosity of foamed gel was low while injecting, but became larger after 15 days of gelation. Foamed gel shows good injectivity performance in porous media.
2. Results of coreflooding show that approximately70% of residual oil in place is recovered after the injection of 0.2–0.3 PV of foamed gel, indicating that foamed gel is a promising agent for enhancing oil displacement efficiency in non-homogeneous porous media.
3. Micromodel visualizations demonstrate the potential of foamed gel for residual oil mobilization. The amoeba effect, Jamin effect and good oil resistance give foamed gel greater mobility control and superior shut-off capacity in porous media compared to conventional aqueous foam.

## Supporting Information

S1 Video This video shows the excellent oil resistance of foamed gel at the present of crude oil. (MPG)

S2 Video This video shows the Amoeba effect I of foamed gel, large foam passes though pore throat by deforming into slender dumbbell-shaped foam. (MPG)

S3 Video This video shows the Amoeba effect II of foamed gel, small foam passes though pore throat by bending deformation. (MPG)

S4 Video If the amount of foams reaches a certain level as the fluid migrates continuously, much of the foam will come together and generate great additional force to crude oil, this additional force can be transformed into displacing force to improve oil recovery. (MPG)
